# Pathogenicity and virulence of *Mycobacterium abscessus*

**DOI:** 10.1080/21505594.2025.2508813

**Published:** 2025-05-26

**Authors:** Soledad Soverina, Haleigh N. Gilliland, Andrew J. Olive

**Affiliations:** Department of Microbiology, Genetics, and Immunology, College of Osteopathic Medicine, Michigan State University, East Lansing, MI, USA

**Keywords:** *Mycobacterium abscessus*, mycobacterial disease, non-tuberculous mycobacterium, host response to mycobacteria, virulence strategies, new models for host–pathogen interactions

## Abstract

Non-tuberculous mycobacteria (NTM), such as *Mycobacterium abscessus* (Mab) are an increasing cause of human disease. While the majority of immunocompetent hosts control Mab infections, the robust survival of Mab within the environment has shaped survival in human cells to help drive persistence and cause inflammatory damage in susceptible individuals. With high intrinsic resistance to antibiotics, there is an important need to fully understand how Mab causes infection, define protective host pathways that control disease, and develop new strategies to treat those at high risk. This review will examine the existing literature related to host–Mab interactions with a focus on virulence, the host response, and therapy development. The goal is to highlight key gaps in our understanding and describe novel approaches to encourage new research avenues that better define the pathogenesis and host response against this increasingly important human pathogen.

## The emergence of Mycobacterium abscessus as a public health concern

The genus mycobacterium, first defined by Lehman and Neumann in 1896, comprises over 200 species [1,2]. These bacteria are acid-fast, non-motile bacilli with a high lipid content that plays an important role in cell wall structure and virulence. Excluding *Mycobacterium leprae* and *Mycobacterium ulcerans*, the genus is divided into two large groups for diagnostic purposes: tuberculosis-causing mycobacteria or non-tuberculous mycobacteria (NTM) [3–5]. Historically, *Mycobacterium tuberculosis* (Mtb), is the most well-known pathogenic mycobacteria responsible for pulmonary and extrapulmonary tuberculosis. In contrast, NTMs cause a broad range of opportunistic infections in humans [6,7]. The number of pulmonary infections caused by rapidly growing NTMs continues to rise, increasing more than 400% from 1987 to 2015 [8,9]. Among the NTMs, the slow growing *Mycobacterium avium* and the rapidly growing *Mycobacterium abscessus* (Mab) are the most clinically relevant with Mab representing 65–80% of NTM lung infections seen in patients with genetic or acquired lung disease [10–12]. This review will focus on our current state of research regarding Mab infection as a guide to better understand NTMs and highlight areas for future studies.

Mab was first isolated from a deep abscess in a patient in 1953 by Moore and Frerichs [13]. Since then, the taxonomy of Mab has been controversial due to similarities with two other species: *M. masiliense* and *M. bolletii*. Using whole genome sequencing on clinical isolates of Mab, Bryant *et al.* distinguished three subspecies that were established as part of the *Mycobacterium abscessus complex* in 2016 [14,15]. The importance of differentiating these three subspecies lies in the success of therapy since *M. abscessus* and *M. bolletii* possess inducible antibiotic resistance mechanisms that are lacking in M. *massiliense* [16]. Thus, genome sequencing and comparisons of the *hsp65, rpoB, and erm* [17] genes are used to differentiate subspecies of the *M. abscessus complex* [18]. Acknowledging these genetic and possible phenotypic differences between strains of the Mab complex, many studies do not distinguish between these subspecies and for simplicity, we will discuss findings throughout this review using Mab as an identifier of any strain within the Mab complex.

## Environmental and host pressures shaping Mab evolution

Unlike Mtb, which is an obligate human pathogen and does not possess an environmental reservoir, Mab is ubiquitous in the environment, being found in both soil and water. A key factor contributing to the persistence of Mab in the environment is the structure of the mycomembrane which includes glycopeptidolipids (GPLs) that provide resistance to external stressors [19–21]. Despite the high energetic cost of synthesis, the mycomembrane allows bacterial adherence to inert surfaces, protection against disinfectants or antibiotics, resistance to desiccation, and contributes to biofilm formation. In line with these characteristics, fomites, and biofilms found on shower heads or medical equipment in hospitals have been largely implicated in Mab transmission [14,22–24].

Given its ubiquitous prevalence in the environment, humans likely encounter Mab regularly. However, frequent human exposure to Mab does not explain its pathogenicity. The genome of Mab displays a collection of elements acquired during its evolution, possibly through interactions with other organisms in aquatic or soil ecosystems [25]. Findings from several studies support the hypothesis that environmental stressors, such as free-living amoebae, “trained” Mab to be opportunistic pathogens, perhaps similar to the evolution of another opportunistic respiratory pathogen *Legionella pneumophila* [26,27]. Comparisons of the intracellular Mab transcriptome in amoebae like *A. castellani* or murine macrophages find similar gene expression patterns reflecting an enhanced stress response and metabolic adaptations to the intracellular lifestyle [28]. This includes the expression of genes like *eis2* that enhance intracellular survival [28]. Thus, environmental interactions likely shaped the pathogenicity of Mab in ways that remain to be fully understood.

Another contributing factor to the evolution of Mab into a pathogen is horizontal gene transfer. Ripoll et al. reported the presence of arsenic and mercury resistance genes in the Mab genome, supporting the hypothesis of horizontal gene transfer and ecosystem selective pressures as contributors to Mab evolution [25]. This is further supported by the identification of a phospholipase c (*plcC)* gene that is similar to *P. aeruginosa* or *C. violaceum* yet distinct from Mtb [29]. Detailed studies on this phospholipase C showed that it is cytotoxic to murine macrophages and is expressed only during intracellular growth in amoebae [29]. Interestingly, preculturing Mab in amoebae results in increased virulence in mice, suggesting important connections between Mab found in the environment and human infection [29].

Despite the presence of full-length prophage and prophage-like elements in the Mab genome, the transformation or transduction mechanisms driving horizontal gene transfer remain unclear [30,31]. What is clear is that horizontal gene transfer events increased Mab infectivity by allowing rapid adaptation to intracellular environments. In 2016, Bryant et al. recognized the first indications that Mab may be emerging as a true pulmonary pathogen [32]. Genomic analysis of Mab clinical isolates from around the globe suggested the possible evolution of Mab towards a true pathogen following a two-stage model [33]. In the first stage, selective pressure from the environment drives the acquisition of genes that increase virulence, then in the second stage, Mab evolution within the host drives diversification and increases pathogenicity in macrophages. Yet this evolution comes with a cost of reduced transmission fitness [33]. The presence of hypermutator Mab strains in the lungs of Cystic fibrosis patients, who are particularly susceptible to Mab infection, is similar to the evolution of other respiratory pathogens including *P. aeruginosa* and *B. cepacia* in Cystic fibrosis patients [34,35]. Thus, both environmental and host pressures are continually shaping the genome of Mab making the genetic diversity of Mab strains incredibly high compared to Mtb [36]. The impacts of this genetic diversity on human disease remain to be fully understood but likely contribute to variability in the disease states observed in humans.

## Diverse human infections caused by Mab

Similar to other NTMs such as *M. avium*, Mab is observed clinically in a variety of infection sites including the respiratory tract, skin wounds, and systemically. Each of these infections likely requires distinct co-factors to enable effective Mab colonization and survival to cause disease.

### Respiratory infections

The majority of immunocompetent individuals exposed to Mab resolve infection rapidly. Yet for patients with underlying pulmonary conditions including cystic fibrosis (CF), chronic obstructive pulmonary disease (COPD), or bronchiectasis, infections with Mab can be life-threatening [11,14,37,38]. The mechanisms for Mab susceptibility in CF patients are exacerbated by hyper-inflammation, the presence of a thickened mucus membrane, and ciliary dysfunction that primarily result from mutations in the Cystic Fibrosis Transmembrane Conductance Regulator (CFTR) gene [39,40]. Loss of CFTR function dysregulates alveolar macrophage phagocytosis and efferocytosis function, resulting in the inability of CF patients to successfully clear bacteria and other debris from their pulmonary space [41]. Patients with COPD and bronchiectasis have chronic airway inflammation and build-up of excess mucus lining the pulmonary cavity [17,37]. The subsequent thickening of their bronchi walls causes insufficient mucociliary clearance and dysfunctional macrophage immune responses [41,42]. In addition, research has linked Mab susceptibility to alveolar macrophage dysfunction resulting from silica exposure [43]. A closer look at the lung environments seen in at-risk patients implicates functional macrophage responses are essential to maintain lung homeostasis and effectively eradicate Mab exposures.

### Soft tissue infections

Contrary to Mab pulmonary infection, skin and soft tissue infections mostly occur in immunocompetent individuals following traumas that allows inoculation of Mab into interstitial tissues. For example, Mab-contaminated medical devices used during surgical procedures are linked to Mab infection, while exposure of wounds to contaminated water or soil can introduce Mab [44,45]. Soft tissue infections have been reported after cosmetic procedures such as breast or gluteal augmentation, liposuction, and mesotherapy [46–48]. Most cutaneous lesions are localized and manifest with cellulitis, erythematous violaceous papules, or nodules. Lesions usually compromise the dermis and can be self-limited or resolved with antibiotic treatment, wound draining, and debridement [45,49]. The number of lesions depends on the immune state of the patient and immunocompromised patients are at a much higher risk of developing disseminated disease [50]. The robust survival of Mab in otherwise sterile environments highlights the need for appropriate sterilization procedures to reduce the risk of possible Mab contamination that can endanger patients.

### Systemic or disseminated disease

Disseminated Mab infections involve lymph nodes, internal organs, and/or positive blood culture [51]. Although disseminated NTM disease is rare, it occurs more frequently in patients on immunosuppressive therapies or patients with primary immunodeficiency including HIV infection; whether this is true for Mab specifically remains unclear [52,53]. Defective cell-mediated immunity is the primary underlying mechanism for high-risk mycobacterial disease in these susceptible individuals [54]. Patients with defects in IFN-γ and IL-12 pathways are predisposed to disseminated NTM disease while HIV patients with low CD4^+^ T-lymphocyte numbers are also susceptible [49]. In addition, patients on long-term immunosuppressive therapies including oral prednisone and anti-TNF therapy show higher risks of Mab infections [50,55]. Disseminated disease in these immunocompromised individuals results in more severe infections and a high mortality rate [51,56]. Even after achieving negative blood cultures following antibiotic therapy, patients with disseminated disease often relapse and die from the infection or complications [51,57]. Thus, better understanding of the correlates of protective immunity against Mab infection is needed to identify patients at the highest risk of severe disease, while early diagnostics are essential to prevent disseminated disease before complications occur.

## Virulence mechanisms contributing to host–Mab interactions

For those susceptible patients, it is critical to understand how Mab persists and acquires essential nutrients to survive and cause disease. The last 20 years have uncovered a range of virulence strategies employed by Mab that manipulate the host cells, drive inflammatory responses, and prevent effective treatment. These virulence mechanisms include modulating surface lipid composition, upregulating transmembrane proteins that can efflux toxic molecules, and expressing secretion systems to modulate the host environment and obtain nutrients.

Central to Mab survival in the environment and to Mab causing pulmonary disease are the outer lipids that can withstand stress while modulating host inflammatory responses. Key regulators of the outer lipid structure are mycobacterial membrane proteins large (Mmpl) that drive the transport of lipids to the surface of Mab [58,59]. Similar to Mtb, Mmpl3 is an essential gene in Mab as a key transporter of trehalose monomycolates that are required precursors of mycolic acids and possible targets for antibiotic therapy [60]. Mmpl8 was shown to be an essential transporter of glycosyl-diacylated-nondecyl-diols and is also required for macrophage survival [61]. In addition, other Mmpl proteins modulate the efflux of antimicrobial compounds and are likely important for survival in both the environment and mammalian hosts [62].

Perhaps, the most important Mmpl for Mab clinical observations is Mmpl4. Mmpl4 regulates the transport of glycopeptidolipids (GPLs), the most abundant lipids on the surface of many Mab strains [63,64]. The presence of GPLs distinguishes strains that have a smooth and glossy colony morphology, while the absence of GPLs gives Mab a rough colony morphology. Key differences in smooth and rough isolates during host–Mab interactions, and from clinical data, suggest similarities to Mtb that express phthiocerol dimycocerosate (PDIM) on their surface to modulate innate immune signaling and resist cell-autonomous responses [65–67]. GPLs on Mab conceal underlying surface components to facilitate and establish infection without driving robust inflammatory responses [68,69]. GPLs consist of a core lipopeptide with an extending fatty acyl chain linked to tripeptide-amino-alcohol core in addition to modifications such as glycosylation [70,71]. During persistent infections, Mab spontaneously stops producing GPLs making a transition from smooth to rough morphology. This transition coincides with increased disease severity that is associated with increased inflammation and the generation of large Mab cords that are not effectively phagocytosed [72]. Rough variants also initiate higher rates of phagosomal rupture and type I IFN signaling cascades, resulting in cell death mediated cell-to-cell transmission [11,73,74]. Even though there are clear differences in smooth and rough variants, the host signals or selective pressures that drive this shift are not well understood [75]. While several mutations in genes such as Mmpl4 that prevent GPL transport are known, there remains a possibility that Mab temporally regulates transcriptional networks controlling GPL expression based on environmental cues such as temperature [63,76–78]. Thus, further research is needed to define the regulation of GPLs, Mmpl4, and their importance on pulmonary disease.

Beyond Mmpls, Mab uses other mechanisms to survive within a macrophage. For example, the transmembrane P-type ATPase, *mtgC*, is induced inside cells to help Mab take up Mg^2+^ inside macrophages [79,80]. However, *mtgC* mutant growth rates remain unaltered during infection, highlighting undefined Mab virulence factors used for essential nutrient uptake [73]. In terms of specialized secretion systems, Mab encodes several type VII secretions systems that contribute to infection and are specific to mycobacteria and limited gram-positive bacteria. The role of type VII secretions systems known as ESX (ESX-I – ESX-V), are well studied in Mtb and are widely implicated in bacterial survival, fitness, and virulence [81,82]. Mab only expresses two analogous secretion systems to Mtb: ESX-III and ESX-IV. Of these, ESX-IV aids in Mab virulence by acquiring essential nutrients in the phagosome and subverting phagolysosome toxic defense mechanisms [83]. Transposon mutagenesis studies found Mab uses an ATPase gene, *eccB4*, in the ESX-IV secretion system to disrupt phagosome acidification [83]. The Mab ESX-IV secretion system may serve a similar function to ESX-I in Mtb since both are required to subvert phagosome maturation events [84]. ESX-III is important for intracellular survival and to cause infection in animals while driving inflammatory responses, yet mechanistic understanding of this secretion system remains relatively limited [85]. In more recent work, it was found that Mab contains Esx-P a secretion system located on episomal plasmids, yet the role of these acquired secretion systems remains almost entirely unknown [86,87]. Thus, while ESX secretion systems play a key role in host–pathogen interactions, the mechanistic function of these systems requires further study.

Similar to Mtb, Mab survives in oxygen replete conditions [88]. Chronic inflammation and mucus build-up seen in the lungs of at-risk patients, phagosome maturation, and granuloma formation all represent oxygen starved host environments where Mab persists [88,89]. The two-component signaling regulon DosRST is a well-studied virulence factor for Mtb pathogenesis and survival in hypoxic conditions [90–93]. Mab has its own, unique DosRS system that is activated in hypoxic, carbon monoxide, and nitric oxide environments [94]. A recent transcriptomic study found that loss of DosRS_Mab_ disrupts more than 200 genes and attenuated bacterial growth in oxygen starved conditions and an increased shift towards the rough morphotype [94]. Corroborating these findings, a recent alveolar organoid model found smooth variants favor traditional biofilm formation to permit Mab survival, while host oxidative stress drove rough Mab variants into a serpentine cording [95].

Complicating matters further, isolated sputa from Mab-infected patients often show co-infection with more than one microorganism, including *Pseudomonas aeruginosa*, *Staphylococcus aureus,* and other NTMs [96–98]. This microbial community that exists in altered lung environments is characterized by frequent and often unrelenting antibiotic therapy. As a result, recent studies have started looking into the role of co-infections in Mab pathogenesis and virulence. One study has shown that *Pseudomonas aeruginosa* inhibits Mab biofilm formation [99,100]. However, when researchers introduced a common antibiotic, Clarithromycin, used to treat both pathogens, they found treatment selectively decreased *Pseudomonas aeruginosa* biofilm development, and increased Mab survival [99,100]. Furthermore, it has been suggested that Mab degrades a *Pseudomonas aeruginosa* quorum-sensing molecule, *Pseudomonas* quinolone signal (PQS), to gain a competitive advantage during co-infection [101,102]. Follow-up experiments examining this antagonistic relationship within liquid-cultured biofilms were unsuccessful in determining the specific interbacterial mechanisms at play [101]. These studies show the virulence potential of Mab to cause disease. While much remains to be learned, the clear consensus is that Mab has acquired and evolved several strategies to survive inside host cells and drive distinct disease states. These disease states are modulated by the host immune response that senses the infection, activates inflammation, and recruits immune cells to the lung environment.

## The host response during Mab infection

### The innate immune response to Mab infection

Early detection and innate responses during Mab infection are fundamental to control pathogen growth and shape disease progression. These early interactions are mediated by the detection of Mab pathogen-associated molecular patterns (PAMPs) by innate immune pattern recognition receptors (PRRs), which results in the production of inflammatory cytokines that activate and recruit other immune cells. These other immune cells target the pathogen but can also drive deleterious tissue damage. Thus, careful regulation of innate immune networks is needed to effectively clear Mab infections from the pulmonary space.

The majority of immunocompetent individuals clear Mab infection suggesting functional innate immune responses effectively limit Mab pathogenesis. Tissue-resident fetal liver-derived AMs in the lungs are the first immune cells to confront respiratory pathogens, and recent studies show the majority of Mab inside AMs following pulmonary infection [103,104]. These immune cells are thought to be less inflammatory to help maintain pulmonary function [105]. However, ongoing infections and disruptions to lung physiology, which occur in susceptible patients, are characterized by increased numbers of myeloid-derived macrophages [106]. The myeloid-derived macrophages are more inflammatory and may change the trajectory of the initial host–Mab interactions. These differences are clearly important, but their relative contribution in the innate response during Mab infection remains unexplored. One hypothesis is that healthy patients that rapidly clear Mab employ AMs while patients with previous lung damage have increased inflammatory myeloid-derived macrophages, that together with changes in lung physiology/structure, may contribute to severe disease. Even though the majority of studies examine innate interactions using macrophage models that may not reflect the physiology of distinct lung macrophage subsets, they are still very useful to define key innate responses against Mab.

**Figure 1.**
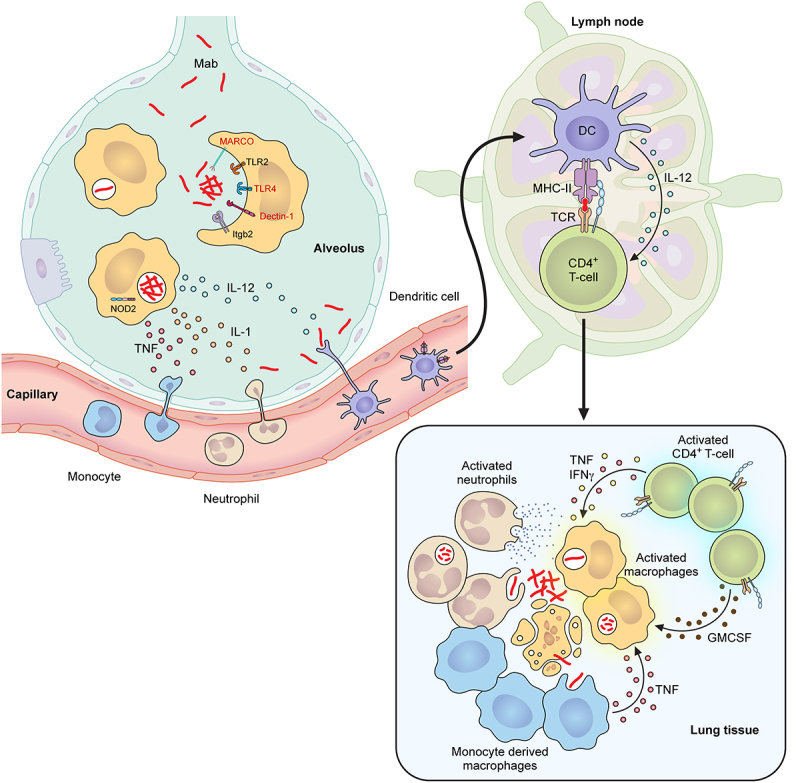
Crosstalk between the innate and adaptive immune response during Mab infection.

Following exposure, Mab is engulfed by macrophages by phagocytosis or receptor-mediated uptake. Initial recognition of pathogens requires effective interactions between Mab and macrophages (Figure 1) [107–109]. This process is initiated by ligand binding to macrophage surface PRRs, including Toll-like receptors (TLRs), Dectin receptors, and scavenger receptors [110–113]. Dectin-1 and TLR2 have been shown to initiate uptake by macrophages during infection [78,114]. Yet, follow-up studies with Dectin-1 deficient mice saw no defects in Mab control compared to wild-type controls, highlighting parallel mechanisms of Mab uptake in macrophages [115]. Other host-associated surface molecules including integrins such as Itgb2, mediate the initial interaction of Mab with macrophages, but the role of these receptors in the lungs remains unclear [116]. For smooth Mab strains with high levels of GPL, many Toll-like receptor ligands are masked, preventing rapid and robust inflammatory responses by the host cells. In contrast, reduced GPL in rough strains exposes PAMPs that interact with TLRs, triggering the production of pro-inflammatory cytokines [78]. TLR2 drives interactions with rough Mab variants to initiate uptake and induce more rapid and robust TNFα expression in macrophages [78]. In addition, two reports suggest that TLR4 may play a role similarly to what is observed with Mtb [117,118]. Thus, the composition of the outer membrane of Mab is a key mediator of innate responses, yet further studies are needed to determine the specificity of distinct innate pathways in different macrophage subsets and against diverse clinical isolates.

Upon exposure, resident alveolar macrophages engulf Mab by binding surface receptors such as TLR2 and Itgb2 (Indicated in Black). Additional receptors (indicated in Red) like TLR4, MARCO, and Dectin-1 may modulate uptake but are more controversial or unknown. Once internalized other PRRs such as NOD2 drive initial cytokine responses. Smooth Mab variants are maintained in “loner” pathogen-containing vacuole while rough variants are “social” resulting in more rapid inflammation. Regardless, the initial innate responses lead to the production of chemokines, IL1, and TNF that recruit monocytes and neutrophils to produce ROS and other antimicrobial mechanisms that may contribute to killing extracellular Mab. In addition, IL-12 drives the trafficking of dendritic cells to activate CD4+ T cells in the lymph node. Activated Th1 cells arrive at the site of infection and contain Mab while activating antimicrobial control by producing IFNγ, TNF, and possibly GM-CSF.

In addition to TLRs, Nucleotide-binding Oligomerization Domain-containing 2 (NOD2) is implicated in Mab-dependent inflammatory responses. This cytosolic PRR is recruited to the pathogen-containing phagosome where it directly senses peptidoglycans released from the bacterial cell wall [119–121]. Activation of NOD2 induces downstream signaling that upregulates antigen presentation and activates transcription factors, such as NF-κB [122,123]. NF-κB translocation to the nucleus induces many inflammatory cytokines and antimicrobial defense mechanisms including the inducible nitric oxide synthase (iNOS) [122,123]. NOD2 deficient mice show delayed Mab clearance due to reduced NO production and bacterial control [124,125]. Given its cytosolic localization, how NOD2 is recruited to the phagosome and differentially senses smooth and rough strains is unknown.

Beyond PRRs, scavenger receptors are critically important in the innate immune response as observed following trehalose dimycolate (TDM) stimulation of murine macrophages [126]. During Mtb infection, the scavenger receptor MARCO together with the co-receptor CD14 and the PRRs TLR2 and TLR4 are required to induce NF-κB activation. TDM is also present in Mab and acts as a virulence factor with immunomodulatory effects [127,128]. However, the specific role of MARCO and CD14 during Mab infection remains unknown. Other immune receptors including MINCLE that recognize TDM in the context of Mtb may play a role during Mab infection, but these also remain to be fully understood [129]. Thus, while Mab shares many common PAMPs with Mtb, our understanding of the initial innate interactions significantly lags behind.

Following internalization, Mab is maintained in a pathogen-containing vacuole and proceeds through the conserved phagosome maturation process. This maturation activates a conserved downstream signaling cascade that leads to the remodeling of the actin cytoskeleton and the formation of the nascent phagosome [130–132]. The phagosome interacts with various endosomal compartments that result in an anti-microbial environment characterized by acidification by vacuolar-ATPases, production of phagocyte oxidase-mediated reaction oxygen (ROS) and nitrogen species (RNS) and delivery of recruited cathepsins and hydrolases [133–136]. For most phagocytosed cargo, the matured phagosome fuses with the lysosome, a compartment containing numerous anti-microbial acid-activated hydrolytic enzymes, creating the phagolysosome [137,138].

Studies of early host–Mab interactions elucidated important aspects of phagosome structure. Smooth Mab strains are maintained in tight phagosomes that contain a single bacterium that is surrounded by a thick GPL layer [72]. In contrast, rough strains that lack GPL are maintained in “social” phagosomes with several bacteria [72,139]. Given the aggregative phenotype of rough strains, these bacteria can stall the phagocytosis process and remain in the phagocytic cup or near the surface of the macrophages. The distinct phagosome environments and the differences in GPL expression for smooth and rough Mab strains are also thought to drive differences in the inflammatory response. Since rough variants remain in social phagosomes, they more readily drive inflammatory cytokines and induce cellular apoptosis that result in cord formation in the extracellular environment [140]. Conversely, smooth strains survive better inside macrophages by preventing phagosome-lysosome fusion, enabling long-term persistence without robust PRR activation signaling. Taken together, differences in the phagosome structure between smooth and rough Mab strains directly alter the innate immune response.

The macrophages that initially sense and respond to Mab infection must contain the infection. This initial containment is likely controlled by the production of TNF by innate immune cells. TNF is an essential cytokine for granuloma formation, maintenance, and bacterial control as demonstrated by studies in TNF knockout mice during tuberculosis infection [141]. While TNF KO mice are susceptible to Mab infection, the contribution of innate cells and adaptive cells to TNF production remains to be dissected [142]. Beyond TNF, lung macrophages are key producers of IL-1 cytokines. IL-1 signaling is well characterized during Mtb and MAC infections [143,144]. *In vivo* models show higher mortality rates in IL1R1^−/−^ mice and mice lacking either IL-1β and/or IL-1α, yet the role of these two IL-1 proteins remains controversial [144,145]. In contrast, the role of IL-1 during Mab remains relatively understudied as only IL-1β deficient animals have been examined and no studies explore deficiencies in IL-1 R or IL-1α. Among published work, several studies suggest that different Mab isolates induce inflammasome activation, partially through robust ROS production, suggesting IL-1 levels are increased during Mab infection [146,147]. In addition, one recent study showed that the loss of IL-1β does not alter bacterial control but does modulate T cell effector function and tissue damage [148]. While IL-1α is known to play a crucial role in initiating and amplifying inflammation by alveolar macrophages, its regulation and function during Mab remains unknown, possibly due to a lack of studies specifically examining AMs during Mab infection [149]. Studies with other respiratory pathogens like *Legionella pneumophila* show that AM-secreted IL-1α initiates and orchestrates the inflammatory response in the lungs of infected mice yet this remains to be examined during Mab infection [150,151]. Altogether, while IL-1 cytokines are likely critical for host–Mab interactions in the lungs, how they differentially control Mab growth and regulate tissue damage will need to be further studied.

Another important family of cytokines that influence the outcome of mycobacterial infection are type I interferons. IFNα and IFNβ play a complex role during bacterial infections as studies show that these type I interferons downregulate proinflammatory cytokines such as TNF and IL-1 and IL-12 and its excessive production increases susceptibility to Mtb in mice [152–154]. *Ex vivo* studies with rough strains of Mab in murine bone marrow-derived macrophages demonstrated that IFNβ production via the CGAS-STING pathway inhibits IL-1β production, thereby facilitating bacterial spread [74,147]. Conversely, another group demonstrated that a rough strain of Mab induces IFNβ production that is TLR2 and TLR4 dependent yet is independent of CGAS-STING and is required for antimicrobial nitric oxide in the lungs [117]. The IFNβ production in Mab-infected BMDMs was also strain-dependent: smooth variants induce little IFNβ production and therefore survive intracellularly, whereas rough strains induce high levels of IFNβ in infected macrophages contributing to bacterial clearance. These conflicting reports surrounding type I IFN suggest that both cell type and strain type are critical determinants of the IFN response by macrophages and must be carefully examined both *ex vivo* and *in vivo*.

One of the most critical cytokines produced by innate immune cells is IL-12 which drives the activation of Th1 CD4+ T cells to produce IFNγ (discussed in detail in the adaptive immunity section below). In 1996, IL-12 was recognized as one of the products during the early innate immune response to infection with *M. tuberculosis* and humans with mutations in IL-12 (IL-12p40) or its receptor (IL-12 Rβ1) are associated with increased susceptibility to mycobacterial disease [155–157]. While studies suggest IL-12 may be critical to control Mab disease, this remains to be fully understood experimentally [158]. Altogether, lung macrophages are a critical determinant of Mab disease or protective immunity.

Following initial Mab interactions with macrophages in the lungs, chemokines, and inflammatory cytokines, drive the recruitment of other inflammatory cells including neutrophils, monocyte-derived macrophages, and dendritic cells [142,159]. Data are conflicting on the role of neutrophils with some studies suggesting they can control Mab while others suggesting less effective killing [160–162]. Further work is needed to define whether neutrophils contribute to initial control or are associated with worse disease outcomes similar to Mtb infection. Beyond neutrophils, dendritic cells likely traffic Mab antigens to draining lymph nodes to initiate T-cell activation and recruit adaptive immune cells to the site of infection. The crosstalk between pulmonary innate immune cells and the adaptive immune response requires further study to define the kinetics and key cell types that initially respond to control Mab-mediated pulmonary disease and activate subsequent adaptive immune responses.

### Adaptive immune responses to Mab infection

Once dendritic cells traffic to the draining lymph nodes, they present Mab antigens directly to naïve T cells that begin to activate, differentiate into distinct effector functions and traffic back to the infected lung environment. While careful kinetic studies related to T cell-mediated activation remain limited, some studies show that in immunocompetent animals there is an influx of activated T cells by 2 weeks following infection [163]. The recruitment of adaptive immune cells coincides with increased bacterial control and eventually leads to granuloma formation that contains phagocytes, T cells, and B cells (Figure 1) [73,159]. While the role of antibodies produced by B cells in protection remains unclear, Mab-specific antibody production is observed consistently [164]. For T cell-mediated immunity, IL-12 and IFNγ are key to controlling Mab infection and disease, and animal models consistently show an important role for CD4+ T cells in the lungs [142,158]. A study in severe combined immunodeficiency (SCID) mice conducted by Byrd and Lyons showed Mab persistence in the lungs of both BALB/c and SCID mice up to 28 d post infection, with SCID mice showing higher disseminated infection in the spleen compared to controls [165]. Similarly, Rottman, et al. showed higher bacterial burden in both the liver and spleen of Rag2 and CD3ε knockout mice, highlighting a particular importance of T-cells in Mab control [142]. More specifically, studies in mice show that pulmonary Mab infection drives the activation of T-helper 1 (Th1) cells that are characterized by the simultaneous production of both IFNγ and TNF [142,159]. The loss of IFNγ and TNF are known risk factors for Mab infection and Rottman, et al. corroborated these results in mice showing higher disseminated bacterial burden in the liver and spleen of IFNγ receptor 1 knockout and TFNα knockout mice [166–168]. Interestingly, IFNγ knockout animals showed delayed bacterial clearance and survived the infection, yet mice lacking TNF succumbed to disease. This suggests two important conclusions. First, there may be several immune effectors that can help control Mab. Second, TNF may play a larger role during Mab control in mice compared to IFNγ, which is different from infections with Mtb. However, balance of these responses likely remains important since during Mtb infection both excessive IFNγ and TNF drive an increase host cell tissue damage and a breakdown in disease tolerance, while limited cytokine release leads to poor immune activation and unrestricted bacterial growth [169–171]. One recent study characterized the cellular response to Mab in C3HeB/FeJ mice that model necrotic lesions observed in humans during Mtb infection [172]. They found that these animals controlled both smooth and rough Mab variants, albeit they observed a significant delay in T cell activation by the smooth strain. These data align with an adult zebrafish study where robust T cell responses were observed during Mab infection with rough variants, but infection with smooth variants were less severe and control was mediated by TNF produced by innate immune cells [173]. This suggests that lower inflammatory responses by smooth variants may directly delay the onset of adaptive immunity, yet future work will be needed to dissect these intriguing mechanistic questions.

The adaptive response against Mab in mice is better understood than in humans where the CD4+ T cell response is less well characterized. Patients with acquired immunodeficiency syndrome are highly susceptible to Mtb and MAC, but fewer Mab infections are observed in these patients [52]. In patients with cystic fibrosis, Th1-skewed T cell responses are observed in patients with a history of Mab infection [174]. Additionally, there is cross-reactivity of T cells against Mab antigens from patients that are BCG-vaccinated or have a history of MAC infection [175,176]. Taken together, there are many open questions regarding the progression of adaptive immune cells and their role in protection or pathology during Mab infection. This includes the specific role of GM-CSF in controlling Mab infection. While mice deficient in GM-CSF signaling are susceptible to Mab infection, the lung physiology is significantly altered in these mice due to the lack of functional alveolar macrophages and the increased build-up of surfactant [177]. During Mtb infection, GM-CSF can help directly control infection in macrophages [178–180]. A portion of this GM-CSF is produced by lymphoid cells including NK cells and CD4+ T cells [178–180]. Yet the cell-type-specific role of GM-CSF and its direct anti-Mab effects remains to be studied. Additionally, Th17 responses during Mab infection are observed and in animal models there are suggestions that a robust Th17 response drives immunopathology [148]. However, one recent study associated decreased IL-17 with susceptibility to pulmonary Mab infection [181]. Altogether, it is clear that CD4+ T cell responses are critically important to control Mab and it is likely that there is a fine balance between protective and pathologic inflammation.

## Therapeutic approaches to treat Mab infection

### Antibiotic therapy of Mab in the face of high resistance levels

**Figure 2.**
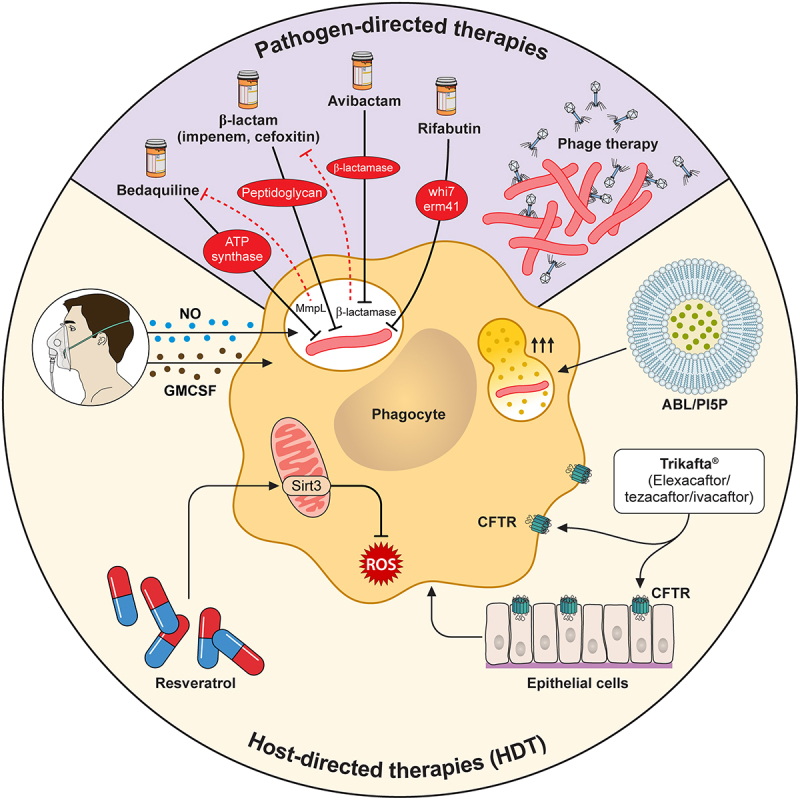
Pathogen- and Host-directed therapies are needed to overcome Mab resistance to antibiotics.

Patients who acquire Mab infection and progress to disease are treated with antibiotics as the frontline standard of care. However, Mab remains incredibly challenging to eradicate once infection takes hold. Even though Mab is rapidly growing compared to Mtb, treatment lengths for Mab can extend beyond those for Mtb passing 6 months or more [182]. A major reason for this extended treatment time is the high rates of resistance of Mab to a variety of approved antibiotics. Mab is intrinsically resistant to many antibiotic classes currently available, including macrolides, aminoglycosides, rifamycins, tetracyclines, and β-lactams (Figure 2) [183–185]. One mechanism of intrinsic resistance is the low permeability of the Mab cell envelope. The high lipid content and thickened mycobacterial cell wall provide effective barriers against hydrophilic and lipophilic antimicrobials and are considered a contributing factor to low permeability [183,186]. With that said, porins within the myco-membrane permit diffusion of hydrophilic antibiotics through the cell envelope [187,188]. These porins act in synergy with Mab-specific antibiotic inducible resistance mechanisms, leading to the upregulation and expression of efflux pumps, antibiotic inactivating enzymes, and target-modifying enzymes [187,188]. Efflux pumps protect bacterium against toxic molecules by exporting toxins and metabolites to the extracellular environment [189,190]. Mab encodes components of an ATP-binding cassette (ABC) transporter family efflux pump. These ABC-type multidrug transporters hydrolyze ATP to pump molecules across the mycomembrane and maintain bacterial homeostasis [25]. However, the dynamic role of Mab specific ABC efflux mechanisms still need exploration. Additionally, Mab MmpLs serve as efflux pumps to transport multiple drugs across the mycomembrane and are known to play a key role in Mtb intrinsic drug resistance [58,61,191–196].

Mab is inherently resistant to several frontline antibiotics through the expression of a β-lactamase, an inducible macrolide resistance system *whib7/erm* [17], and inducible efflux pumps (MmpL/S). To overcome these mechanisms, combination therapies that target both the pathogen and host may be required to eradicate infections. First, drugs that overcome inherent resistance such as Avibactam and Rifabutin, which inhibit β-lactamase and *erm* [17] induction, respectively, may enable other drugs like Bedaquiline, Macrolides, and β-lactams to be more effective. Additionally, directly targeting Mab with Phage may overcome widespread antibiotic resistance. Amplifying the host response and modulating the host environment is a parallel treatment strategy used in combination with antibiotics. These therapies include direct inhalation of antimicrobial effectors like GM-CSF and Nitric Oxide (NO) as well as drugs that target cellular metabolism and lysosome fusion such as Resveratrol and ABL/PIP5 liposomes. Recent approval of Trikafta, that improves the underlying physiological defects in CF patients to reduce mucus in the lungs, is also an important new host therapy that alters the permissive lung environment.

Because different Mab strains are differentially antibiotic resistant, there is no FDA approved drug regimen for Mab pulmonary infection [12]. This is not only due to variable antibiotic resistance but also the variable host environments seen in patients with genetic or acquired lung diseases, including CF and COPD. Oftentimes, these patients face a relentless infectious cycle with one or more antibiotic-resistant strains. As a result, effective antibiotic therapies are guided by patient isolate antibiotic susceptibility testing. Currently, the most common antibiotics used to treat pulmonary Mab infection include two or more intravenous drugs (amikacin, tigecycline, imipenem, and/or cefoxitin) combined with one or two oral antimicrobials (clofazimine, linezolid, and/or azithromycin) for 3 months [12,197]. These treatment timelines are often extended, and current estimates suggest that only 50% of treatment regimens achieve successful clearance within the first round of therapy [12]. Importantly, while several macrolides such as Clarithromycin and Azithromycin can initially be effective, due to Mab inducible macrolide resistance these antibiotics can become rapidly ineffective against a subset of strains. This mechanism is mediated by WhiB7, a multidrug-inducible transcriptional activator that modulates a large set of Mab genes during infection, including *eis2* and *erm41* [198–200]. Antibiotic resistance studies have confirmed a single nucleotide exchange in *erm41* is responsible for abrogating macrolide resistance (Figure 2) [201,202]. This polymorphism is present in 15–20% of clinical isolates, highlighting the continued requirement for extended antibiotic susceptibility testing for all clinical isolates prior to treatment as *erm41*-mediated resistance is only observed after several days [203,204].

Other antibiotics show some efficacy against a variety of Mab clinical isolates. These include the ATP synthase inhibitor Bedaquiline, a recent addition to the Mtb treatment program, that is effective in a variety of animal models and the clinic [182,205]. However, mutations in the Mab transcriptional repressor TetR, MAB_2299c modulate Mab-specific MmpL efflux pump expression, leading to increased Mab resistance to clofazimine and Bedaquiline [192]. Rifabutin, a Rifampin analog, was also recently shown to have efficacy against Mab infections [206–208]. One potential advantage of Rifabutin is that this drug may suppress inducible macrolide resistance by preventing the expression of *whiB7* and *erm(41*) [209]. A similar approach to prevent the induction of antibiotic resistance is also taking shape for treating Mab infections with other antibiotics with inherent resistance. Mab hydrolyzes many β-lactam antibiotics, such as imipenem and cefoxitin, by expressing a β-lactamase, Bla_Mab_ [210–212]. However, the recent development of β-lactamase inhibitors such as avibactam provide a new opportunity to more effectively employ β-lactam antibiotics which will control persistent Mab infection (Figure 2) [210]. A recent report showed that Mab treatment costs can amount to $50,000 USD, making the financial burden of treatment an important factor to consider [182]. Clinical trials to develop more effective therapies are underway, with inhaled liposomal amikacin being a promising candidate [213]. All things considered, as with any prolonged treatment timeline, patient compliance, and antibiotic-related toxic side effects continue to complicate treatment options for many at-risk patients thus increasing the need for new approaches to therapy.

### Host-dependent effects of antibiotic treatment

Antibiotics interfere with the immune system either indirectly by disrupting the body’s natural microbiota, or directly by modulating host immune cell function [214,215]. These interactions impact treatment efficacy and general host susceptibility to infection. Adverse side effects of antibiotic treatment following Mab infections are well known, with almost 78.8% of patients reporting ototoxicity, gastrointestinal distress, and myelosuppression [182]. As a result, nearly half (48.3%) of patients require treatment plan modifications prior to treatment completion [12,182]. The majority of reported adverse side effects are associated with tigecycline, linezolid, and amikacin treatment [182]. Unfortunately, these three antibiotics are correlated with the highest treatment success rates compared to other, better tolerated antibiotics. Therefore, research focused on understanding the host impact of antibiotic treatment is of critical importance to develop more tolerable treatment strategies.

### Host-directed therapies to augment Mab control

The challenges associated with antibiotic therapy to treat Mab infections are driving researchers and clinicians to examine alternative treatments including host-directed therapies (HDTs). HDTs are promising for Mtb strains with multidrug resistance and function to activate host protective immune responses without exacerbating inflammatory damage. For Mtb, many HDTs are effective including drugs targeting autophagy, lipid metabolism, and cell death cascades [216]. A similar logic holds with Mab infections where overcoming high levels of antibiotic resistance may require leveraging the host immune response (Figure 2). Mab may persist in macrophages because it is not efficiently delivered to lysosomes following phagocytosis. Thus, drugs that drive increased phagolysosome formation including the delivery of phosphatidylinositol 5-phosphate (ABL/PI5P) show increased immune control of Mab infection in both human and mouse macrophages [217]. Further studies using drugs that alter intracellular trafficking should continue to be examined.

Similar to Mtb infections, Mab infections are known to alter the cellular metabolism [218]. Thus, targeting metabolic networks in macrophages is a possible HDT. Blocking oxidative phosphorylation of macrophages results in better Mab control while blocking Sirtuin 3 with resveratrol improves Mab disease in both murine and zebrafish models of infection [219]. While resveratrol is a small molecule HDT that targets metabolic pathways, other HDTs include those that activate target cytokines, the delivery of cytokines or antimicrobial molecules directly to the lungs (Figure 2). Recent work suggests that modulating IL17 signaling and angiogenesis/VEGFR may have therapeutic benefits [148,220]. Additionally, the efficacy of inhaled GM-CSF was examined in CF patients with recurrent Mab infection [221]. The findings were encouraging, suggesting aerosolized GM-CSF is a possible therapy for patients with challenging Mab infections. In addition, aerosolized nitric oxide was used in a compassionate treatment of a patient with CF, who tolerated the treatment and showed reduced Mab levels by PCR [222]. While direct aerosolization of cytokines and antimicrobial effectors may have deleterious effects on a broader population, their efficacy in a subset of patients should motivate further studies to identify HDTs that activate similar immune programs.

One of the most important developments for CF patients at risk for Mab infection are new therapies like Trikafta (ETI) that manage the underlying defects caused by CFTR mutations (Figure 2) [223]. While only approved in 2020, clinical reports suggest that patients on ETI may also clear persistent Mab infections. In one case report, a patient eliminated a persistent 12-year Mab infection that was non-responsive to antibiotic therapy [224]. Even more encouraging data werefound from a larger multicenter study that observed that 66% of CF patients with ongoing Mab infections successfully eradicated their Mab infection [223]. These incredible results highlight that the pulmonary environment is a key driving force of persistent Mab infection in the lungs. As further results emerge from CF patients on ETI therapy, there is continued hope that many patients in this highly susceptible cohort will be spared from pulmonary Mab infection.

### Phage therapy as an emerging therapeutic option

In the face of increased antibiotic resistance and a resurgence in phage biology there is increased interest in using phage as an alternative therapy to control Mab infections (Figure 2). Many recent studies were initiated after the report of the successful use of mycobacteriophage to treat a 15-year-old lung transplant patient with disseminated disease [225]. This patient was on antibiotic therapy for persistent Pseudomonas and Mab co-infections for over 8 y. Amazingly, this patient survived the phage therapy, with no severe side-effects and no Mab was isolated from the sputum and serum. The phage therapy consisted of a cocktail of three engineered phages that were administered intravenously two times daily over the course of 32 weeks. More recent work on phage/Mab interactions has better defined host/Mab/Phage interactions that may influence pathogenesis and therapy. In one report it was shown that phages can lower intracellular burdens of Mab [226]. In another set of reports from the Kremer research group, it was shown that the Mab-specific phage Muddy can synergize with antibiotics and help control Mab infection in a CFTR zebrafish model, but only if the innate immune system is intact [227]. In follow-up studies, they found that Muddy and the phage BPs both require trehalose polyphleates (TPPs) to successfully infect Mab [228]. Furthermore, the loss of surface TPPs in Mab through the loss of the transporter Mmpl10, results in decreased intramacrophage survival and disease in a zebrafish model [229]. Altogether, these results highlight an important host/Mab/phage interaction that can help to better target key aspects of Mab pathogenesis for new therapies.

### Vaccine development to prevent Mab infection

Ultimately, long-term protection against Mab will require the development of vaccines that prevent persistence and lung damage. Effective vaccines against any mycobacterial species remain elusive. For Mab, it still remains unclear whether memory responses are robustly induced as there is a paucity of clinical evidence to suggest prior colonization/infection with Mab protects against infection. Even so, one key question with potential Mab vaccine development is whether immunization with *Mycobacterium bovis* – BCG results in cross-protection against Mab. While BCG is compulsory to protect children from Mtb meningitis in many areas of the world, this vaccine as currently formulated and delivered does not provide robust protection against pulmonary disease in adults [230]. While altering the immunization delivery does better protection in mice and non-human primates against challenge with Mtb, the effectiveness of this approach against NTMs remains unclear [231,232]. One cohort study found that children immunized with BCG had far fewer NTM infections, but the durability of this protection is unknown [233]. One mechanistic study from humans who were PPD positive or voluntarily were immunized with BCG showed that T cells from these patients cross-reacted with Mab and MAC antigens [175]. These cross-reactive T cells controlled intracellular Mab and were capable of robustly producing IFNγ. Studies in mice corroborated these findings, and further suggested that BCG vaccinations drive broader cross-reactivity of T cells against Mab than Mtb infection [175]. One additional study vaccinated mice with the Mtb vaccine ID91, that is a fusion of four Mtb antigens and a TLR4 activating adjuvant, then challenged these mice with the NTM *Mycobacterium avium* [234]. Some protection was observed but not to the same extent as BCG vaccination. Thus, while much research is examining the utility of BCG and other Mtb vaccines by modulating the site of vaccination and formulation, it must be considered how these approaches may drive more broad immunity against NTMs such as Mab.

Unfortunately, few studies have worked to develop new protective vaccine candidates against Mab infection. This major gap in research will require a much more detailed understanding of the protective immune pathways that prevent deleterious lung disease while also identifying key protective antigens that drive effective immune responses. To address antigen identification Dar et al. used an in-silico approach to predict protective antigens that would be effectively presented in MHC alleles [235]. This analysis identified four antigens with secreted or cell wall localization and included resuscitationpromoting factor (RPF) and the invasion protein Inv1. Yet whether these targets actually activate protective T cell responses is unknown. A separate set of studies has examined whether Mab genes that are associated with intracellular survival in macrophages, including MgtC and PLC, could be developed into vaccines to protect against Mab [80,236]. Using a DNA vaccination strategy, these studies found robust antibody responses against Mab that reduced infection. While antibodies are likely to help augment effective immunity, better understanding the T cell antigens that drive effective CD4+ T cell responses during Mab infection will need to be explored in detail in the future. Overall, while a protective vaccine against Mab would likely protect those patients at highest risk of disease, there remains a major need for a fundamental understanding of protective immunity and antigens that drive T cell responses during Mab infection in order to make this a reality.

### The future of Mab pathogenesis research: Model systems and tools to dissect virulence and test drug/vaccine efficacy

While much has been discovered about Mab host–pathogen interactions over the last 20 y, many fundamental questions about pathogenesis, protective immunity, and drug development remain. Answering these questions will require using a variety of model systems that will dissect key mechanisms of protection and disease.

#### Cellular and ex vivo models

Understanding host–Mab interactions at the cellular level requires models of pulmonary cells that interact with Mab in the lung environment. Given the known importance of macrophage–Mab interactions, many studies use macrophage cell lines including RAW cells and THP-1 cells [147,237,238]. These have been informative for characterizing a variety of phenotypes during Mab infection but have several caveats that are common with immortalized cells lines including differences in inflammasome activation. For primary cells, bone marrow-derived macrophages (BMDMs) and human monocyte-derived macrophages (hMDMs) are often used due to ease of access to high quantities of cells [239–242]. Both hMDMs and BMDMs enable larger scale *ex vivo* experiments to identify immune networks activated during Mab infection. The increased genetic tractability of these cells allows important host genetic studies to characterize host/Mab interactions [240,243,244]. One recent study took advantage of human pluripotent stem cells to derive human macrophages and characterized Mab interactions [245]. Robust infection and growth of Mab was seen in these cells, inflammatory cytokines including IL-8 were induced, and intracellular killing by antibiotics was observed. This novel approach can now be employed to understand a wide range of host–Mab interactions in myeloid-derived macrophages.

While clearly an important cellular model, myeloid-derived macrophages are functionally distinct from the first immune cells encountered by Mab in the airways, the alveolar macrophage (AMs). There remain few studies that directly examine how AMs sense and respond to Mab infection or characterize distinct replication dynamics in these cells compared to BMDMs. One reason for this lack of data is the challenges associated with studying primary AMs. From mice, very few cells can be isolated from bronchial lavage, and in standard culture conditions, primary AMs lose their characteristic phenotypes and functions [246]. Recent advances in culturing murine AMs *ex vivo* present an opportunity to better characterize differences in bacterial control and inflammation in these lung resident cells. This includes *ex vivo* AMs (exAMs), murine *ex vivo* AMs (mexAMs) and fetal liver-derived alveolar-like macrophages (FLAMs) [246–248]. Importantly, these approaches present an opportunity to genetically manipulate AMs in ways that were previously impossible, including conducting forward genetic screens. In parallel to these murine AM cells, the recent development of a human alveolar-like macrophage (HAMs) model enables these studies to be completed in human cells as well [249]. Beyond macrophages, recent work studying human lung epithelial cells grown in an air–liquid interface model enables better understanding of Mab growth in the lung environment using reductionist approaches [250]. Taken together, there are many new opportunities to examine host–Mab interactions in macrophages and epithelial cells that better model the cellular milieu in the lungs. Leveraging these models will enhance our understanding of how lung immune cells contribute to Mab infection, disease, and protection.

### Non-mammalian in vivo models: Amoeba

Given the environmental prevalence of Mab, interactions with unicellular eukaryotic organism likely occur on a regular basis. Several investigations leverage tractable amoeba including *Acanthamoeba castellanii* and *Dictyostelium discoideum* [61,238]. These model organisms uncovered key genes required for Mab intracellular survival, including MMPL8, and are used to screen the effectiveness of antimicrobial compounds. Comparing similarities and differences between amoeba and macrophages is likely to identify key mechanisms regulating how Mab senses and adapts to an intracellular lifestyle and avoids cell-autonomous killing.

#### Zebrafish and Drosophila

Several studies use the zebrafish (*Danio rerio*) model as a tool to examine granuloma formation and host responses in an intact animal. The genetic tractability is a key advantage of this model as mutations, such as CFTR that is associated with CF patients can be easily modeled [41]. Since zebrafish embryos are translucent, they are amenable to live imaging studies that can monitor the initiation of infection, immune cell recruitment, and survival of the animals all in a single experiment [41,251,252]. Furthermore, this model is useful to test drug efficacy in animals in a rapid and high throughput manner. More recently, an adult zebrafish model was developed enabling Mab persistence to be examined in addition to using host reporter strains and genetics to track key interactions in the animals [173]. Beyond zebrafish, *Drosophila melanogaster* are used to examine immune-mediated control of Mab infection providing new mechanistic insights to the host–pathogen interaction [253,254]. Thus, there are several non-mammalian model systems that continue to shed light on critical interactions between Mab and the host and are likely to inform mechanisms driving disease or protection far into the future.

#### Mammalian in vivo models

As highlighted throughout this review, mice continue to be an important model system to examine Mab–host interactions. However, distinct strains of mice produce distinct infection kinetics and disease progression. Immunocompetent strains, including C57BL/6 mice, do not progress to disease and are only transiently infected [163]. It could be argued that this is an excellent model of most humans, that likely become exposed to Mab yet do not progress to significant clinical disease. However, genetic knockouts on C57BL/6 background are an important tool to dissect the contribution of distinct immune pathways, including key cytokines that are required for pulmonary clearance and host survival [142,177]. While many Mtb studies leverage more susceptible strains such as C3HeB/FeJ mice, these mice are not as susceptible to Mab infection suggesting key differences in the manifestation of Mtb and Mab pulmonary disease [172,255]. Immunocompromised mice, including SCID mice infected with Mab, develop more severe infections and are useful for drug efficacy studies but given the dysregulated host responses may not be useful to characterize key host–pathogen interactions [177]. More recently, genetically diverse mice, such as those in the collaborative cross, have been used to dissect variability in responses to pathogens including Mtb, but these models have yet to be systemically examined during Mab infection [256,257]. Using genetically diverse animals may identify genetic backgrounds that model distinct aspects of Mab infection and disease similar to Mtb where different CC strains withstand or succumb to disease with variable bacterial levels.

Modifications to the host and bacteria in immunocompetent mice can drive more robust and human-like disease in this model. One recent study found that pretreatment of animals with corticosteroids results in more robust colonization and disease pathology [258]. Alternatively, embedding Mab in agarose beads was shown to alter infection sites within the lungs perhaps serving as a better model of patients with ongoing lung disease [259,260]. Agarose bead infections showed more robust infections and disease pathology, suggesting important connections between the anatomical location of Mab in the lungs and disease progression.

### Bacterial and host genetic tools

Beyond models to study distinct aspects of Mab disease, improvements in molecular genetic approaches on both the host and pathogen are helping to better define key host–Mab interactions. Genome-wide transposon mutagenesis studies identified key virulence factors as well as essential genes required for Mab growth *in vitro* such as the PBP-lipo, which is involved in cell wall synthesis and antibiotic sensitivity [83,261,262]. More targeted CRISPR-based approaches are being used in Mab to enable controlled expression of key virulence determinants. For example, one study disrupted the *mps1* gene associated with the production of GPL in the outer membrane [263]. A more recent version of this approach enables rapid gene destruction in Mab using a specialized dual-plasmid system [264]. To better understand key gene regulation of Mab may require the use of transcriptional reporters that are broadly used in Mtb in addition to inducible gene expression systems such as Tet-OFF [36]. Given the low cost of genome sequencing, examining phenotype differences between large numbers of clinical isolates and defining important genetic changes is now possible. These approaches have been used to identify new regulators of virulence and antibiotic resistance in addition to understanding the plasticity of the Mab genome [36,265].

It is clear that our understanding of how Mab infection is controlled mechanistically by the host remains limited. No studies to date systematically examine host restriction factors that regulate Mab intracellular growth and persistence, leaving many open questions remaining. Recent advances in host genetic manipulation using CRISPR-Cas9 present a unique opportunity to better define these pathways and understand new vulnerabilities of Mab. As an example, a genome-wide CRISPR-Cas9 knockout library in immortalized BMDMs was recently used to identify host factors required for Mab uptake into cells [116]. By coupling bacterial fluorescent reporters with a host-based forward genetic screen a new understanding of how host integrins are regulated on the surface of cells to mediate Mab uptake was uncovered. Coupling these host-based genetic approaches with bacterial genetic approaches will enable the systematic use of genetics squared to dissect important interactions between the host and pathogen.

## Looking to the future

Altogether, Mab infections will continue to present a challenge to clinicians in the absence of further drug, therapy, and vaccine development. It is likely that exposures to Mab and infections with Mab will only continue to increase with a changing landscape of world demographics, as populations skew towards the elderly who are more immunocompromised with existing lung damage. Thus, it is imperative that we continue to understand the fundamental biology surrounding host–pathogen interactions during Mab infection. As highlighted throughout this review, while we have a cursory understanding of some protective host responses and virulence tactics, our understanding lags significantly behind in comparison to Mtb infections. Thus, future efforts must seek to fill these important gaps to define what drives protective immunity against Mab infection, work to identify patients who are at the highest risk of disease and develop new therapies to help these patients.

## Data Availability

No data was generated as part of this review article.
